# Assessment of Health Benefits and Cost-Effectiveness of 10-Valent and 13-Valent Pneumococcal Conjugate Vaccination in Kenyan Children

**DOI:** 10.1371/journal.pone.0067324

**Published:** 2013-06-24

**Authors:** Philip Ayieko, Ulla K. Griffiths, Moses Ndiritu, Jennifer Moisi, Isaac K. Mugoya, Tatu Kamau, Mike English, J. Anthony G. Scott

**Affiliations:** 1 Kenya Medical Research Institute/Wellcome Trust Programme, Nairobi, Kenya; 2 Department of Global Health and Development, London School of Hygiene and Tropical Medicine, London, United Kingdom; 3 Kenya Medical Research Institute/Wellcome Trust Programme, Kilifi, Kenya; 4 Division of Vaccine and immunizations, Ministry of Public Health and Sanitation, Nairobi, Kenya; 5 Nuffield Department of Paediatrics, University of Oxford, Oxford, United Kingdom; 6 Nuffield Department of Clinical Medicine, University of Oxford, Oxford, United Kingdom; 7 Department of Infectious Disease Epidemiology, London School of Hygiene & Tropical Medicine, London, United Kingdom; University of California, San Francisco, United States of America

## Abstract

**Background:**

The GAVI Alliance supported10-valent pneumococcal conjugate vaccine (PCV10) introduction in Kenya. We estimated the cost-effectiveness of introducing either PCV10 or the13-valent vaccine (PCV13) from a societal perspective and explored the incremental impact of including indirect vaccine effects.

**Methods:**

The costs and effects of pneumococcal vaccination among infants born in Kenya in 2010 were assessed using a decision analytic model comparing PCV10 or PCV13, in turn, with no vaccination. Direct vaccine effects were estimated as a reduction in the incidence of pneumococcal meningitis, sepsis, bacteraemic pneumonia and non-bacteraemic pneumonia. Pneumococcal disease incidence was extrapolated from a population-based hospital surveillance system in Kilifi and adjustments were made for variable access to care across Kenya. We used vaccine efficacy estimates from a trial in The Gambia and accounted for serotype distribution in Kilifi. We estimated indirect vaccine protection and serotype replacement by extrapolating from the USA. Multivariable sensitivity analysis was conducted using Monte Carlo simulation. We assumed a vaccine price of US$ 3.50 per dose.

**Findings:**

The annual cost of delivering PCV10 was approximately US$14 million. We projected a 42.7% reduction in pneumococcal disease episodes leading to a US$1.97 million reduction in treatment costs and a 6.1% reduction in childhood mortality annually. In the base case analysis, costs per discounted DALY and per death averted by PCV10, amounted to US$ 59 (95% CI 26–103) and US$ 1,958 (95% CI 866–3,425), respectively. PCV13 introduction improved the cost-effectiveness ratios by approximately 20% and inclusion of indirect effects improved cost-effectiveness ratios by 43–56%. The break-even prices for introduction of PCV10 and PCV13 are US$ 0.41 and 0.51, respectively.

**Conclusions:**

Introducing either PCV10 or PCV13 in Kenya is highly cost-effective from a societal perspective. Indirect effects, if they occur, would significantly improve the cost-effectiveness.

## Introduction

In the year 2008, approximately 8.8 million deaths occurred worldwide in children less than five years and pneumococcal disease caused an estimated 521,000 of these deaths. [1], [2] Trials of pneumococcal conjugate vaccines (PCV) conducted among infants have shown significant efficacy against invasive pneumococcal disease (IPD), [3], [4], [5], [6] and by early 2010 PCV had been introduced into routine immunization programmes in over 50 high- and middle-income countries. [7], [8] The World Health Organization had also recommended the introduction of PCV into the immunization schedules in developing countries with high background rates of childhood mortality. [9].

In 2011, the GAVI Alliance received pledges for sufficient funding to support introduction of PCV into all countries with Gross National Income per capita below US$ 1,520. [10] Kenya introduced universal infant vaccination with 10-valent PCV in February 2011 with financial support from the GAVI Alliance. [11] Published country-specific estimates of the cost-effectiveness of PCV are scarce for African countries. [12], [13], [14] The existing studies do not include indirect effects of pneumococcal vaccination, and most adopt a narrow health provider perspective. Some international groups have produced global models, largely based on variations in infant mortality. [15] Convincing analyses of cost-effectiveness from developing countries require local data on incidence and serotype distributions and effectiveness estimates that go beyond mortality. [16].

In this economic evaluation we use Kenyan data on both treatment costs and pneumococcal disease incidence by syndrome and serotypes together with African evidence of vaccine efficacy to estimate the cost-effectiveness of delivering PCV10 in routine immunization services in Kenya. We include the vaccine impact against both morbidity and mortality and also explore the incremental benefits of incorporating indirect effects and of switching to 13-valent vaccine (PCV13).

PCV10 is currently available to Kenya at a cost of $3.50 per dose, which is being co-financed by the GAVI Alliance and the Kenyan government for a 10-year period. [17] Donor funds, availed under the Advance Market Commitment (AMC), are being used to pay an extra $3.50 per dose for approximately 20% of all vaccine doses purchased during this period. [18] At the end of the co-financing period the government will take over the full cost of the vaccine. The purpose of this cost-effectiveness analysis is to assist policy makers in Kenya to plan beyond the co-financing period. The study may also assist other countries with similar vaccine costs and pneumococcal disease patterns, and inform funding decisions of international vaccine donors.

## Methods

The analysis was conducted from a societal perspective considering both medical and non-medical costs and productivity losses. A static decision analytic model (as opposed to dynamic disease transmission model) using aggregate-level data was employed in calculating health benefits, costs and incremental cost-effectiveness of vaccinating all infants born in Kenya in 2010 at current vaccination coverage. We converted all costs to 2010 US$ using the GDP deflator. [19].

The base case analysis compared the discounted (3% per year) costs and effects of universal PCV vaccination to those associated with no PCV introduction and included only direct vaccine effects. We assigned probability values to all costs and events and calculated the expected values of each decision option. The assigned probability values were identified from a variety of data sources, including published literature identified through structured searches of PubMed, reviewing relevant health ministry’s records, and expert opinion where data were unavailable from the first two sources (Table 1).

**Table 1:** Parameters used in the base case analysis for pneumococcal vaccination in Kenyan infants born in 2010 *BCV- Base case value.

Parameter	Base case	Reference	Statistical distribution	Source
**IPD incidence (**per 10^5^ child-years)				
* Meningitis*				
0–11 months	48.3	Kilifi HDSS	Poisson (48.3)	Kilifi HDSS
12–23 months	22.4		Poisson (22.4)	
24–59 months	5.0		Poisson (5)	
* Sepsis*				
0–11 months	27.4	Kilifi HDSS	Poisson (27.4)	Kilifi HDSS
12–23 months	27.2		Poisson (27.2)	
24–59 months	13.4		Poisson (13.4)	
* Bacteraemic pneumonia*				
0–11 months	74.0	Kilifi HDSS	Poisson(74)	Kilifi HDSS
12–23 months	41.6		Poisson(41.6)	
24–59 months	17.8		Poisson(17.8)	
* Non-bacteraemic pneumonia*				
0–11 months	972.9	Extrapolation fromCutts [4]	NA	Extrapolation from Cutts et al
12–23 months	592.9		NA	
24–59 months	235.4		NA	
**Vaccination**				
*PCV9 efficacy against serotype specific disease*				
Meningitis	92%	Cutts [4]	Beta(92,8)	Cutts et al
Sepsis	92%		Beta(92,8)	
Bacteraemic pneumonia	77%		Beta(77,23)	
Non-bacteraemic pneumonia	77%	Extrapolated fromCutts [4]	Beta(77,23)	Extrapolation from Cutts et al
**Access to healthcare**				
Outpatient care	37%	Kallander [23]	Beta (16,28)	Kallander et al
Inpatient hospital care	40%		Beta(18,26)	
**Health outcomes**				
Major post discharge meningitis sequelae	25%	Edmond [27]	Beta (25,75)	Edmond et al
*Case fatality with hospital care*				
IPD (sepsis, bacteraemic pneumonia)	28.2%	Berkley, [41] expert opinion	Beta (28,72)	Berkley et al, expert opinion
Non-bacteraemic pneumonia	5.7%	Berkley, [41] expert opinion	Beta (6,94)	Berkley et al, expert opinion
*Case fatality without hospital care*				
Meningitis	97%	Expert opinion	Beta (97,3)	Expert opinion
Sepsis and bacteraemic pneumonia	50%		Beta (4,4)	
Non-bacteraemic pneumonia	12%		Beta (12,88)	
**Inpatient treatment costs**				
Meningitis	$357.74	Ayieko [28]	Gamma (4,19)	Ayieko et al
Sepsis or pneumonia	$74.64		Gamma (1,314)	
**Outpatient treatment cost**				
Childhood fever	$2.74	Larson [29]	NA	Larson et al
Discounting				
Costs = 0%; effects = 0%	NA	Assumption	NA	
Costs = 3%; effects = 3%	NA	Assumption	NA	

NA- Not applicable.

### Disease Incidence

We classified pneumococcal disease into invasive (IPD) and non-invasive disease. IPD was defined by the isolation of *S. pneumoniae* from blood, cerebrospinal fluid (CSF) or pleural fluid. We sub-classified IPD into three syndromes; meningitis, sepsis, and bacteraemic pneumonia, and included only one non-invasive pneumococcal syndrome; non-bacteraemic pneumonia. The possible outcomes from sepsis, bacteraemic- and non-bacteraemic pneumonia are death or survival with complete recovery while for meningitis they are death, survival with complete recovery or survival with sequelae.

Hospital IPD incidence rates were calculated using the results of paediatric inpatient surveillance from Kilifi District Hospital, Kenya. [20] All children admitted to this hospital undergo blood cultures on admission, and those meeting specific criteria undergo CSF or pleural fluid cultures. [21] The hospital is situated at the centre of the Kilifi Health and Demographic Surveillance System (KHDSS), which covers an area of 891 km^2^ and re-enumerates a population of about 250,000 people three times a year through household visits. Computer systems at KDH link paediatric admissions and microbiological results to the population register of the KHDSS. The hospitalised incidence is given each year as the number of cases of IPD residing within the KHDSS divided by the mid-year population of the KHDSS. [21] Data from 7 years (2003–2009) were used to estimate mean annual rates. Since hospital-based surveillance underestimates the true burden of pneumonia and meningitis by 45% and 30% we adjusted the incidence rates, [22] dividing them by the proportion of children who access hospital care. These proportions were derived from a Ugandan study of health seeking behaviour (Table 1). [23].

We divided the IPD incidence into fractions attributable to meningitis, sepsis and bacteraemic pneumonia on the basis of clinical data from KDH. Meningitis was defined as positive CSF or blood culture and CSF white cell count ≥50×10^6^ cells/L or a ratio of CSF glucose to plasma glucose less than 0.1. Presentation with fast breathing plus a history of either cough or difficult breathing was required for a pneumonia diagnosis, chest indrawing was required for severe pneumonia and one or more of hypoxia, cyanosis, inability to drink, prostration or convulsions for very severe pneumonia. IPD cases not meeting meningitis or pneumonia criteria above were defined as sepsis. [24] We estimated the incidence of non-bacteraemic pneumococcal pneumonia preventable by vaccination by extrapolating results of the vaccine efficacy trial in The Gambia: in this trial the vaccine-preventable burden of radiologically-confirmed pneumonia was 7.5 times greater than the vaccine-preventable burden of IPD. [4] Therefore, to derive the incidence rate of non-bacteraemic pneumococcal pneumonia we multiplied the observed IPD incidence in Kilifi by 7.5 to estimate the incidence of radiologically-confirmed pneumococcal pneumonia and then subtracted the reported incidence of bacteraemic pneumococcal pneumonia to avoid double counting.

We used the efficacy estimates of PCV9 from a trial in The Gambia because this setting resembles Kenya epidemiologically. [4] Following the criteria of non-inferiority of immunogenicity used by the regulatory agencies for licensing higher valent vaccines, [25] we assumed that the efficacy of additional antigens in the 10- and 13-valent PCV against their target serotypes was equal to that in the baseline 9-valent PCV. To project the number of cases preventable in each year of age we multiplied the vaccine efficacy against IPD of serotypes in the vaccine in The Gambia trial (92% for meningitis or sepsis, 77% for bacteraemic and non-bacteraemic pneumonia), [4] with the number of cases that were estimated to have disease caused by serotypes contained in the PCV10 or PCV13 vaccine, and multiplied this by immunization coverage. The proportion of IPD that was caused by serotypes in each of the two vaccines was estimated for each year of age from 215 episodes of IPD reported among children under five in Kilifi District Hospital between 2003 and 2009.

### Disability Adjusted Life Years (DALYs) Estimates

The DALYs averted as a result of pneumococcal vaccination were calculated using the formula recommended by Murray et al. [26] The calculations incorporated age weights (0.04), and discounting (0.03). The disability weights were 0.28 for bacteraemic- and non-bacteraemic pneumonia and 0.616 for meningitis. [26] Due to the absence of standard disability weights for pneumococcal sepsis we used the pneumonia disability weight. For lifelong meningitis sequelae, we assumed that 25% of all PCV meningitis cases in Africa develop major neurological deficits, [27] and applied the disability weight for motor deficits (0.334) to these episodes. [26].

### Vaccine Programme

Infant vaccines are delivered through the Kenya Expanded Programme on Immunization (KEPI) currently being run by the Ministry of Public Health and Sanitation’s (MOPHS) Division of Vaccines and Immunizations (DVI). Prior to PCV introduction, seven antigens were routinely delivered during infant immunization thorough KEPI, namely birth BCG, oral polio vaccine (birth, 4, 6 and 10 weeks), DPT plus Hepatitis B antigen and *Haemophilus influenzae* type B (administered as a combined pentavalent vaccine at 4, 6, and 10 weeks). Introduction of pneumococcal vaccine (also administered at 4, 6, and 10 weeks) required strengthening of existing immunization services. In the present analysis capital costs for Expanded Programme on Immunization (EPI) were confined to additional investments in cold chain storage capacity at the national and regional EPI stores across the country. These expenditure data were obtained from DVI and from plans for cold chain procurement specified in the country application for GAVI Alliance support. Initially, Kenya planned to introduce PCV7, which requires 59.9 cm^3^ of storage volume per dose and the cold chain was expanded to accommodate this. A fraction of this substantial cold chain investment was allocated to programme cost estimates for the two PCV formulations in proportion to their smaller storage volumes, being 4.8 cm^3^ for PCV10 and 12.0 cm^3^ for PCV13.

Recurrent costs included vaccines and supply procurement. The current three-dose infant PCV schedule was incorporated in the model and a vaccine wastage rate of 15% applied for PCV and a wastage of 5% for injection and safety equipments. We estimated the price of a single dose of pneumococcal vaccine at US$ 3.50, which is the maximum price the Kenya Government could pay under the AMC contract, which is effective until 2020. Vaccine wastage (15%), freight (2%), and vaccine administration charges were added to the costs per dose. Additional analyses were undertaken to capture the government perspective by applying the co-financing payment of US$0.20 per dose currently paid by the Kenyan government under the GAVI Alliance policy.

### Health-care Seeking Behaviour

There are widespread disparities in health care seeking behaviour in developing countries. Findings of a recent verbal autopsy study for fatal pneumonia estimated that 37% and 40% of childhood pneumonia cases access outpatient care and inpatient hospital care, respectively and 23% of children do not reach care. [23] Such findings have important implications for the analysis approach we adopted. For example, there is significant potential for underestimating the true pneumococcal disease incidence because the modelled estimates of pneumococcal disease burden are derived from hospital based surveillance yet over 45% of pneumonia cases and 30% of meningitis cases are missed by hospital surveillance because of difficulties in accessing hospital care. [22] We assumed 23% of children do not access care and 37% access out-patient care only as reported in verbal autopsy studies. [23] We presented the uncertainty in the access to care parameters by a beta 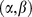 distribution (Table 1). Further, children accessing formal healthcare were assigned lower case fatality rates [beta 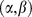] for the different pneumococcal disease syndromes compared to those who failed to reach care (Table 1).

### Pneumococcal Disease Costs

The average cost of inpatient treatment were obtained from a study of hospital resource utilization among 572 paediatric admission at 7 different hospitals in Kenya. [28] The societal cost of treating an episode of inpatient pneumonia or sepsis is US$74.64 (SD 37.23) and an acute meningitis episode cost US$357.74 (SD 335.12) (Table 1). These included direct medical costs, the opportunity cost of caretaker time and household out-of-pocket costs. Outpatient visit costs were obtained from a study on the cost of treating childhood fevers in Kenya. [29]. The costs of treating meningitis sequelae were excluded from the analysis.

### Sensitivity Analysis

Probabilistic sensitivity analysis was used to account for parameter uncertainty by specifying variables as distributions and conducting 10 000 Monte Carlo simulations using Crystal Ball® software (Decisioneering, USA). The stochastic variables identified in the model along with their distributions are presented in Table 1. Results of sensitivity analysis were presented as contribution to variance showing the percentage of variance in the cost effectiveness estimates contributed by each assumption. The contribution to variance is calculated in Crystal Ball by squaring the rank correlation coefficients (between every assumption and every forecast of cost effectiveness while simulation is running) and normalising them to 100%. [30] During simulations the proportions of patients accessing care and pneumococcal case fatality rates were sampled from beta distributions and a Poisson distribution was applied to pneumococcal disease incidence. Conversely, costs were sampled from log-normal distributions. Prediction intervals around the incremental CER were derived from 10 000 Monte Carlo simulations. To incorporate indirect effects we included additional parameters for both herd immunity and serotype replacement. These modifications to the base case model allowed us to assume a “mature” vaccination programme in which indirect effects are established and to model costs and effects for the entire Kenyan population. In the analysis of the entire population, adult age group differences in pneumococcal disease incidence and serotype coverage were accounted for by dividing the population into strata (5–12, 13 to 54, 55 to 64 and ≥65 years).

We projected the magnitude of indirect protection using effectiveness data from the USA following the introduction of PCV7 into the routine vaccination schedule. The magnitude of indirect effects is heterogeneous across different countries, [31] however, we selected these data because the population under surveillance is large and the duration of surveillance is relatively long. In the USA cases of IPD among children aged <5 years declined by 93.6% between 1999–2003 and the vaccine’s direct effect accounted for a 63.9% reduction (vaccine efficacy_×_immunization coverage) in disease burden. [32] The residual reduction (93.9–63.9 = 30%) was caused by indirect protection. We defined the indirect vaccine effectiveness as the proportion of cases of vaccine-type (VT) disease that are not prevented by direct vaccine effects in different age groups but are, however, prevented by indirect vaccine effects. Given the direct vaccine effectiveness estimate above (63.9%) the proportion of cases of VT disease not prevented by the direct effect is 36.1% (i.e. 1–0.639) and the indirect vaccine effectiveness is 83.1% (i.e. 0.300_÷_0.361). We thus multiplied the number of VT cases remaining, after applying direct protection from PCV10 vaccination, with 83.1% to estimate the additional effect of indirect protection and undertook a probabilistic sensitivity analysis within the range 40% to 100%.

We accounted for the negative effect of serotype replacement disease using the same data from the USA. [33] In this surveillance, children aged <5 years were 1.21 times more likely to get non-vaccine type disease after PCV introduction. As there is considerable heterogeneity in the magnitude of serotype replacement disease, geographically we set ranges of 1.0 and 2.4 around this parameter representing settings where no replacement disease occurs and those with very high rates of replacement disease. [31].

## Results

### Disease Impact

A total of 1,407,000 infants born in Kenya in 2010 contributed 6,680,436 child-years of observation during the first five years of life. The model projects a 42.7% reduction in childhood IPD episodes from 96,387 to 55,197 following PCV10 introduction (Table 2). Without PCV vaccination a total of 104,118 all-cause child deaths were projected during follow up based on current under-five mortality rate of 74 deaths per 1,000 births. The model estimated that 14,412 (13.8%) of these deaths were attributable to pneumococcal disease and PCV10 directly prevented 6,358 (6.1%) childhood deaths. The impact of pneumococcal vaccination on mortality caused by meningitis, sepsis, bacteraemic pneumonia and non bacteraemic pneumonia are presented in Table 2.

**Table 2:** Estimated cost and disease impact of pneumococcal vaccination with PCV10 and PCV13 in Kenyan infants born in 2010.

	Without PCV10	PCV10 Introduction	PCV13 Introduction
*Pneumococcal disease episodes* *			
Meningitis	3,180	1,659	1,308
Sepsis	3,449	1,626	1,229
Bacteraemic pneumonia	6,221	3,602	3,027
Non-bacteraemic pneumonia	83,537	48,310	40,497
Total disease episodes	96,387	55,197	46,061
Total reduction in disease episodes	–	41,190	50,326
*Pneumococcal deaths* *			
Meningitis	2,275	1,186	934
Sepsis	1,448	684	576
Bacteraemic pneumonia	2,612	1,513	1,270
Non-bacteraemic pneumonia	8,077	4,671	3,916
Total pneumococcal deaths	14,412	8,054	6,636
Total reduction in pneumococcal deaths	–	6,358	7,776
*Pneumococcal vaccination costs (2010 US$)*			
Pneumococcal vaccine procurement cost	–	$13,662,205	$13,662,205
Injection equipment cost (syringes, safety boxes)	–	$360,306	$360,306
Annualized cold chain expansion costs	–	$14,758	$70,714
Total costs of vaccination	–	$14,037,268	$14,093,224

* The number of pneumococcal deaths and episodes are non-discounted.

### Incremental Costs

Pneumococcal vaccine costs amount to US$ 13,662,205 annually (Table 2). The largest cold chain investments were two cold rooms installed at the national level and three cold rooms at the regional level, at a total cost of US$ 305,000, as well as 800 refrigerators for the lower storage levels purchased for US$1,205,750 (Table 3). The two-dose vial presentation of 10 valent vaccines only requires a fraction of this capacity (Table 3) and therefore incurs only a fraction of cold chain expansion costs. Annual treatment cost averted amounted to US$ 1,958,907 following PCV10 introduction and US$2,397,906 with PCV13 (Table 4).

**Table 3:** Cold chain storage costs incurred by the Kenyan government during 2008 in preparation for pneumococcal vaccine introduction.

	Quantity	Total annualised cost* ($)	Cost allocated to PCV10 ($)	Cost allocated to PCV13($)
*National vaccine store*				
Cold rooms	2	14,302	1,150	5,510
Stand by generators	2	2829	227	1,090
*Regional level*				
Cold rooms	3	21,453	1,725	8,265
*District stores*				
District storage refrigerators	200	35,338	2,841	13,614
*Immunizing facilities*				
Immunizing facility refrigerators	600	106,013	8,524	40,843
Vaccine carriers	1,000	991	80	382
Cold boxes	50	2,622	211	1,010
Total costs		183,548	14,758	70,714
Cost per child born in 2010		0.13	0.010	0.05
Cost per fully immunized child		0.16	0.013	0.06

**Table 4:** Incremental cost effectiveness of pneumococcal vaccination in Kenya and sensitivity analysis of the impact of indirect effects for PCV10 and PCV13.

	Vaccine costs(US $)	Treatment costs (US $)	Net costs(US $)	Pneumococcal cases	Pneumococcal deaths	Discounted DALYs	Cost (US$) per case averted (95% CI)	Cost per (US$)per deathaverted (95% CI)	Cost (US$) per DALY averted (95% CI)
*Base case(0–59 month old children only and no indirect effects)*									
No pneumococcal vaccination	17,100,167	4,578,815	21,678,982	93,217	13,947	459,145			
With PCV10	31,137,435	2,609,271	33,746,705	53,629	7,831	256,301			
Increment	14,037,764	−1,969,545	12,067,723	39,588	6,116	202,844	300 (145–488)	1,958 (913–3,425)	59 (26–103)
With PCV13	31,193,391	2,167,382	33,360,773	44,780	6,460	211,052			
Increment	14,093,224	−2,411,433	11,681,790	48,437	7,487	248,094	238 (110–390)	1,558 (665–2,764)	47 (20–83)
*Indirect effects in the entire population*									
No pneumococcal vaccination	84,863,269	30,173,792	115,037,061	512,708	102,134	3,957,704			
With PCV10	154,916,931	13,854,396	168,771,327	199,064	49,662	2,053,280			
Increment	70,053,662	−16,319,396	53,734,266	313,644	52,472	1,904,425	189 (85–320)	1,158 (585–2,225)	32 (14–55)
With PCV13	154,972,887	10,886,054	165,858,941	138,968	40,030	1,729,744			
Increment	70,109,618	−19,287,738	50,821,880	373,740	62,104	2,227,960	147 (62–258)	888 (366–1,597)	25 (10–44)

* All costs, pneumococcal episodes and deaths are discounted at an annual rate of 3%.

### Cost-effectiveness

In the PCV10 base case analysis, assuming no indirect effects, a 3% discount rate and a price of US$ 3.50 per dose, the cost effectiveness ratio for PCV10 versus status quo was US$59 (95% CI, 26–103) per DALY averted, US$ 300 (95% CI, 145–488) per case averted and US$ 1,958 (95% CI, 866–3,425) per death averted (Table 4). Working with the same assumptions, cost effectiveness of PCV13 versus status quo was US$ 47 (95% CI, 20–83), US$ 238 (95% CI, 110–390) and US$ 1,558 (95% CI, 665–2,764) per DALY, case and death averted, respectively. The non-discounted estimates of base case vaccine cost effectiveness for both PCV10 and PCV13 are presented as additional material (Table S1).

The Kenya Government currently pays $0.20 per dose under a co-financing arrangement with GAVI. From the Government perspective, introduction of either PCV10 or PCV13 is cost saving. The break-even prices for PCV10 and PCV13 are US$ 0.41 and US$ 0.51, respectively. Accounting for vaccine indirect effects increases the break-even prices to US$ 0.72 for PCV10 and US$ 0.87 for PCV13.

### Sensitivity Analysis

**Figure 1:**
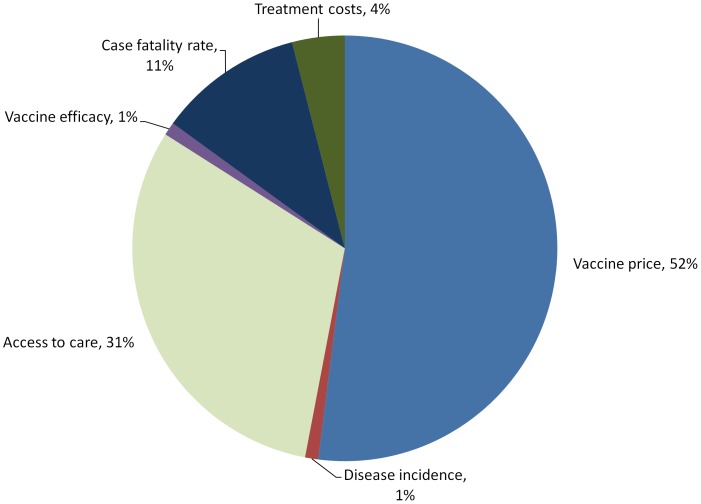
Percentage contribution to variance of uncertain parameters in base case analysis (cost per DALY averted for PCV10).

The cost effectiveness ratios were robust to changes in most of the variables included in the sensitivity analysis providing strong evidence that pneumococcal vaccination would be cost effective. The results of the probabilistic sensitivity analysis are presented in Figure 1. In the base case analysis three factors contributed approximately 94% of the total variance in costs per DALY averted. Vaccine price per dose explained approximately half of the variance in vaccine cost effectiveness. Access to care parameters accounted for most of the remaining variance accounting for 31% of the total variation while assumptions on case fatality of various pneumococcal syndromes also explained a substantial proportion (11%) of reported variation in cost effectiveness. These values are important considering that case fatality rates are significantly higher among children who do not reach formal health care compared to those who do.

Assuming that indirect effects are similar to those observed in USA, pneumococcal vaccination with PCV10 and PCV13 would cost US$32 (14–55) and US$ 25 (10–44) per DALY averted, respectively, representing a 56% improvement in cost effectiveness. The cost per case averted reduces to US$ 189 (85–320) and US$ 147 (62–258) for PCV10 and PCV13, respectively (Table 4). Under these assumptions it would cost US$ 1,158 (505–2,025) and US$ 888 (366–1,597) to avert a single pneumococcal death with PCV10 and PCV13 (48% improvement in cost effectiveness).

## Discussion

This study conducted around the period of PCV introduction in several African countries, and shortly after PCV introduction in Kenya, shows that pneumococcal vaccination is highly cost-effective using Kenya’s current GDP per capita of US$775 as a benchmark. [34] At a cost of US$ 59 ($Int 114) per DALY averted pneumococcal vaccination compares favourably with the most cost effective packages of child health interventions delivered at high coverage in sub-Saharan Africa. Other cost-effective interventions include micronutrient fortification ($Int 19 per DALY), and the incremental inclusion of measles vaccination ($Int 29–58 at different population coverages), pneumonia case management ($Int 73), micronutrient supplementation ($Int 85) and oral rehydration therapy ($Int 106–243 at different population coverages). [35] Data from this region indicates that various HIV/AIDS antiretroviral regimens cost between $Int500 and $Int5000 per DALY averted. [36].

The projected cost effectiveness of pneumococcal vaccination in our study ($Int 114) is slightly higher than the estimates of $Int 100 and $Int 69 per DALY averted among children in GAVI eligible countries and the countries with Under-5 mortality rates of 100–150 per 1000 births, respectively. [15] These differences are not significant because the international economic evaluation in GAVI eligible countries used a vaccine price of $Int 5 compared with a value of approximately $Int 7 (US$3.50) in the present study. In contrast to the international study, [15] our model estimated the epidemiological burden of pneumococcal disease directly from country-specific data. We projected that 14,412 deaths occur during the first 5 years of life among infants born in Kenya during 2010 due to pneumococcal disease. This figure is similar to the estimate of 16,433 deaths among Kenyan children under 5 years during the year 2000 reported in the Pneumococcal Global Burden of Disease (GBD) Study, which relied heavily on published data from Kilifi. [37] The reported improvement in child health indices during the ten-year period between the studies could explain the similarity in mortality despite the annual increase in the size of the Kenyan birth cohort. At the level of morbidity, the GBD study estimated an annual burden of 235,522 cases per year, substantially greater than the 96,387 estimated in our -analysis; the bulk of these differences lies in the category of non-bacteraemic pneumonia for which it is difficult to obtain consistent syndromic data but the comparison illustrates the essentially conservative nature of the parameters selected for our analysis. [38], [39].

Of significance among the assumptions made in the base case analysis is the exclusion of indirect vaccine effects, failure to account for transmission dynamics of pneumococcal disease. However, inclusion of indirect effects in the sensitivity-analysis resulted in an approximately 2-fold improvement in cost-effectiveness ratios. In this sensitivity analysis we modelled the positive impact of herd effect and the negative impact of replacement disease, but did not model further positive gains resulting from, for example, reduced antibiotic resistance. It is likely that the cost-effectiveness ratios presented in the indirect effects scenarios still represent conservative estimates. [16] However, the magnitude of indirect effects in our setting remains uncertain and further studies will be required to refine the estimates after these indirect effects are established.

The cost-effectiveness model indicates that using PCV13 instead of PCV10 would reduce the cost per DALY averted from USD 59 to USD 47 (Table 4). This differential is entirely a function of the relative frequency in Kenya of serotypes 3, 6A and 19A, which are contained in PCV13 alone, compared to the 10 serotypes common to both vaccines. There is no information available on the relative effectiveness of each vaccine against vaccine-type disease nor the relative capacity of each vaccine to cause indirect protection or serotype replacement disease. Furthermore, the model does not incorporate potential protective effects of the Protein D carrier, which is unique to PCV10, against H. *influenzae* disease. Until further epidemiological information becomes available the relative merits of the two formulations will remain uncertain.

The findings of the probabilistic sensitivity analysis confirm the over-riding importance of vaccine price, [15] and access to care estimates in cost-effectiveness analysis and particularly for access to care this sensitivity analysis agrees with previously published epidemiological models of disease burden based on hospital data.^16^ We incorporated the impact of access to care on pneumococcal disease outcome by assigning poorer outcomes (higher case fatality rates) to children with limited access care while those accessing care had lower case fatalities. However, the assumption of similar vaccination coverage in children with and without access to hospital care is a potential shortcoming of our analysis. Our assumption is supported by studies conducted in rural African settings that show that distance to hospital (as a proxy of access to care) does not affect vaccination coverage in children within the hospital’s catchment area. [40] Despite this evidence, in the scenario that children with low access to care also have lower vaccination coverage which is particularly likely in settings with lower health facility density it is plausible that vaccine cost effectiveness will be less favorable. The magnitude of decline in cost effectiveness is likely to be insignificant in areas with functional immunization programmes.

The uncertainty that derives from inaccuracy in access to care parameter indicates the priority for accurate and reproducible field data on health seeking behaviour in the region. Beyond the base case analysis, the parameters for herd protection and replacement disease required for models incorporating pneumococcal disease transmission dynamics are a source of considerable uncertainty and will remain so until these effects have been quantified in a developing world setting.

Our objective was to provide an analysis that would inform decision makers in Kenya, and other similar developing countries. The analysis suggests that introduction of PCV10 in Kenya will reduce the number of cases of pneumococcal disease by 41,190 and reduce childhood mortality by 6.1%. Our base-case estimate of US$ 59 per DALY averted means that PCV is a highly cost-effective intervention whether compared against the benchmark of per capita GDP or against other competing health interventions. Furthermore, this is a highly conservative estimate and realistic adjustment for indirect vaccine effects throughout the whole population improves the cost-effectiveness ratios by approximately 56% (US$ 32 per DALY averted). This analysis provides strong support for the economic case for the introduction and maintenance of the pneumococcal conjugate vaccine programme in Kenya and in other developing countries with similar economic and epidemiological profiles.

## Supporting Information

Table S1 **Non-discounted incremental cost effectiveness of pneumococcal vaccination in Kenya.**
(DOCX)
