# Supplementary material for: Assessment of Health Benefits and Cost-Effectiveness of 10-Valent and 13-Valent Pneumococcal Conjugate Vaccination in Kenyan Children
Source: PLoS One. 2013 Jun 24;8(6):e67324. doi: 10.1371/journal.pone.0067324 (PMC3691111; doi:10.1371/journal.pone.0067324)
Supplement: Table S1 — Non-discounted incremental cost effectiveness of pneumococcal vaccination in Kenya. (DOCX) [file pone.0067324.s001.docx]

Table S1: Base case non-discounted analysis of PCV10 and PCV13 vaccine cost-effectiveness among infants born in Kenya in 2010

|  | Vaccine costs  (US $) | Treatment costs  (US $) | Net costs (US $) | Pneumococcal cases | Pneumococcal deaths | DALYs | Cost (US$) per case averted (95% CI) | Cost per (US$) per death averted (95% CI) | Cost (US$) per DALY averted  (95% CI) |
| --- | --- | --- | --- | --- | --- | --- | --- | --- | --- |
| *Base case( 0-59 month old children only and no indirect effects)* | | | | | | | | | |
| No pneumococcal vaccination | 17,100,167 | 4,728,817 | 21,828,984 | 96,390 | 14,411 | 1,008,870 |  |  |  |
| With PCV10 | 31,137,435 | 2,682,631 | 33,820,066 | 55,199 | 8,054 | 563,157 |  |  |  |
| Increment | 14,037,268 | -2,046,186 | 11,991,082 | 41,191 | 6,357 | 445,713 | 287  (141-465) | 1,881  (852-3,309) | 27  (12-47) |
|  |  |  |  |  |  |  |  |  |  |
| With PCV13 | 31,193,391 | 2,226,656 | 33,420,047 | 46,063 | 6,637 | 463,730 |  |  |  |
| Increment | 14,093,224 | - 2,502,161 | 11,591,063 | 50,327 | 7,774 | 545,140 | 228  (104-377) | 1,487  (631-2,648) | 21  (9-38) |
|  |  |  |  |  |  |  |  |  |  |
